# Surface Modification of Nanoporous Anodic Alumina during Self-Catalytic Atomic Layer Deposition of Silicon Dioxide from (3-Aminopropyl)Triethoxysilane

**DOI:** 10.3390/ma14175052

**Published:** 2021-09-03

**Authors:** Ana Silvia González, Víctor Vega, Ana Laura Cuevas, María del Valle Martínez de Yuso, Víctor M. Prida, Juana Benavente

**Affiliations:** 1 Departamento de Física, Facultad de Ciencias, Universidad de Oviedo, E-33007 Oviedo, Spain; vmpp@uniovi.es; 2 Laboratorio de Membranas Nanoporosas, Servicios Científico-Técnicos, Universidad de Oviedo, E-33006 Oviedo, Spain; vegavictor@uniovi.es; 3 Unidad de Nanotecnología, SCBI Centro, Universidad de Málaga, E-29071 Málaga, Spain; analaura.cuevas@uma.es; 4 Servicios Centrales de Investigación, Universidad de Málaga, E-29071 Málaga, Spain; mvyuso@uma.es; 5 Departmento de Física Aplicada I, Facultad de Ciencias, Universidad de Málaga, E-29071 Málaga, Spain; j_benavente@uma.es

**Keywords:** nanoporous alumina structure, atomic layer deposition, surface modification, APTES and ozone precursors, XPS, impedance and optical measurements

## Abstract

Changes associated to atomic layer deposition (ALD) of SiO_2_ from 3-aminopropyl triethoxysilane (APTES) and O_3_, on a nanoporous alumina structure, obtained by two-step electrochemical anodization in oxalic acid electrolyte (Ox sample) are analysed. A reduction of 16% in pore size for the Ox sample, used as support, was determined by SEM analysis after its coverage by a SiO_2_ layer (Ox+SiO_2_ sample), independently of APTES or O_3_ modification (Ox+SiO_2_/APTES and Ox+SiO_2_/APTES/O_3_ samples). Chemical surface modification was determined by X-ray photoelectron spectroscopy (XPS) technique during the different stages of the ALD process, and differences induced at the surface level on the Ox nanoporous alumina substrate seem to affect interfacial effects of both samples when they are in contact with an electrolyte solution according to electrochemical impedance spectroscopy (EIS) measurements, or their refraction index as determined by spectroscopic ellipsometry (SE) technique. However, no substantial differences in properties related to the nanoporous structure of anodic alumina (photoluminescent (PL) character or geometrical parameters) were observed between Ox+SiO_2_/APTES and Ox+SiO_2_/APTES/O_3_ samples.

## 1. Introduction

Atomic Layer Deposition (ALD), formerly known as atomic layer epitaxy, is nowadays a well-stablished technique allowing for the deposition of smooth and pinhole-free thin films of several materials, namely oxides, nitrides, and sulfides, among others [1,2,3]. Furthermore, ALD enables the deposition of conformal coatings even in three-dimensional, high aspect ratio nanostructured substrates, while keeping an accurate control on the thickness and stoichiometry of the deposits [4]. These features arise from the self-limited nature of the ALD technique, in which reaction kinetics are controlled by surface chemistry, rather than by mass transport phenomena [5]. From all the above reasons, ALD constitutes an outstanding deposition technique, being specifically suitable for surface coating of nanoporous structures, such as nanoporous anodic alumina or polymeric track-etched membranes [4,6,7,8]. Depending on the material of choice for the ALD deposition, different features of nanoporous membranes can be tuned or improved. In fact, nanoporous alumina structures (NPASs) or membranes (NPAMs) obtained by electrochemical anodization of aluminium (two step anodization method [9,10]) consisting in a parallel array of cylindrical pores with narrow pore radius distribution and without tortuosity, whose geometrical parameters (pore size, interpore distance and pore length) can be selected depending on the electrochemical anodization conditions have demonstrated to be adequate supports for ALD deposition of one or two layers of different ceramic oxides, thus enabling to modify their pore-size/porosity and physicochemical characteristics (surface chemistry, selectivity, electrical and diffusive properties) as well as optical characteristics (refraction index, light transmission,…) [7,8,11,12]. Lately, NPASs have been considered as very good candidates to develop chemical/biological sensors due to their biocompatibility (which can be improved by a given coating layer) and high aspect ratio, which favours the enhancement of optical signals by pore-walls modification with specific molecules [13,14].

On the other hand, silicon dioxide is a material of particular interest for membrane modification due to its dielectric nature, high chemical and thermal stability. In this context, silica-alumina composite membranes synthesized through chemical vapor deposition (CVD) of tetraethylorthosilicate (TEOS) and aluminium tri-sec-butoxide precursor have been proposed for application in membrane reactors due to their high gas permeation and selectivity [15] The ALD deposition of conformal SiO_2_ films from a wide variety of silane precursors, including tetraethoxysilane, tris[dimethylamino]silane, bis[diethylamino]silane, (*N,N*-dimethylamino)trimethylsilane, vinyltrimethoxysilane, trivinylmethoxysilane, tetrakis(dimethylamino)silane, tris(dimethylamino)silane, tetrakis(ethylamino)silane, aminodisilane and 3-aminopropyltriethoxysilane (APTES) has been explored in the literature, [16,17,18,19,20,21,22]. This work focuses on the thermal ALD deposition process based on the self-catalytic reaction between 3-aminopropyltriethoxysilane, water, and ozone as precursors. The main advantages of this SiO_2_ deposition approach, based on APTES precursor, lies in the fact that it shows a high degree of thickness control and remarkably good surface coverage even into nanoscale pores, which can be achieved at low growth temperatures of 120–200 °C [22]. Also, APTES precursor is noncorrosive, liquid at room temperature, with sufficient vapor pressure under moderate heating, and commercially available at low cost [23]. Consequently, ALD of SiO_2_ offers exceptional deposition conformality with high aspect ratio even for nanoporous structures like alumina templates [23,24]. In addition, application of APTES modified polymeric membranes and APTES functionalized alumina monoliths for the immobilization of molecules (biotin, rabbit IgG,...) or effective purification of immunoglobulins, respectively, have already been reported [25,26,27,28], opening the field of application of APTES-modified NPAMs.

**Figure 1:**
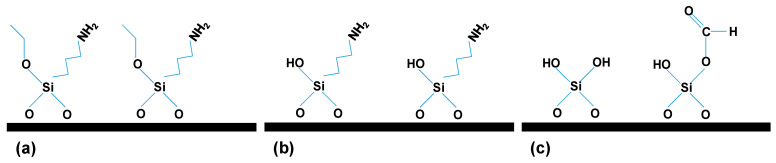
Schematic drawing of the surface reactions taking place during the different steps in a SiO_2_ ALD deposition cycle: (**a**) APTES cycle; (**b**) H_2_O cycle and (**c**) O_3_ cycle.

Another important aspect is that the ALD of SiO_2_ is a three-step reaction sequence based on the subsequent exposure of substrates to APTES, water, and ozone (O_3_), as it can be observed in Figure 1, where it is possible to see that in the first step APTES is chemisorbed onto the surface of the substrate material (Figure 1a), after this, during the H_2_O cycle, the aminopropyl groups existing in APTES catalyze the hydrolysis of ethoxy ligands, leaving the surface terminated with OH groups (Figure 1b). In addition, during the O_3_ cycle, occurs the combustion of the remaining aminopropyl ligands, enabling the production of combustion gases such as CO, CO_2_, H_2_O, and NO_x_. As it was already indicated [29] among these by-products, CO reacts with surface OH groups to form monodentate formates and SiH on the surface (Figure 1c). Finally, the monodentate formates with SiH groups, along with the OH groups, produced during H_2_O exposure, serve as the reactive sites for APTES chemisorption, thus completing the entire ALD cycle [22,23,29]. As a result, surface functional groups change during the different steps that take place during a SiO_2_ ALD deposition cycle, ranging from etoxy, amino and hydroxide groups. This causes that the same material may have slightly different surface groups, which in turn may affect its physicochemical and/or transport properties.

In this work, we analyzed the chemical change caused in a nanoporous alumina structure after being modified with a SiO_2_ layer by ALD technique, which exhibits two different surface terminations (with amino or monodentate formates groups), as indicated in Figure 1a,c, as well as the changes that such terminations provokes in different parameters of interest for sample applications. These changes have been studied by means of several characterization techniques such as scanning electron microscopy (SEM), X-ray photoelectron spectroscopy (XPS), electrochemical impedance spectroscopy (EIS) and diverse optical techniques (photoluminescence, transmittance and spectroscopic ellipsometry measurements), in order to have a complete characterization of the modified samples.

## 2. Materials and Methods

### 2.1. Materials

The nanoporous alumina structure (NPAS) used as support was synthesized by the well-stablished two step anodization method [9,10]. In brief, high purity (99.999%) Al foils (SMP, Barcelona, Spain) with a thickness of 0.5 mm were cut into discs of 25 mm in diameter, thoroughly cleaned by sonication in isopropyl and ethyl alcohols and electropolished in a mixture of perchloric acid in ethanol (1:3 vol.) at 5 °C under an anodic voltage of 20 V, applied between the Al foils and a platinum counter-electrode. Afterwards, the aluminum substrates were rinsed in ultrapure water (18.2 MΩ·cm) and submitted to a first anodization process in a mechanically stirred 0.3 M oxalic acid electrolyte, kept at 0–3 °C by an external recirculating bath. An anodization voltage of 40 V measured versus a Pt counter-electrode was applied by a computer controlled DC power supply (Keithley 2400 sourcemeter, Keithley Instruments, Inc., Cleveland, OH, USA) for 24 h, in order to promote the growth of a self-ordered nanoporous alumina structure [9,10].

The nanoporous oxide layer grown during the first anodization step was selectively etched in an aqueous mixture of CrO_3_ and H_3_PO_4_, at room temperature for 48 h, thus leading to a nanostructured Al surface which retains a regular arrangement of dimples left by the pore bottoms during the first anodization step. A second anodization step, performed under the same electrochemical conditions as the first one is then carried out, the duration of which was adjusted to 33 h in order to obtain a nanoporous alumina sample with around of 60 µm in thickness, according with the oxide growth rate of approximately 1.8 µm/h which has been experimentally measured. The remaining Al substrate was selectively removed by exposing the backside of the samples to CuCl_2_ and HCl aqueous solution, whereas the alumina barrier layer occluding the pores bottom was dissolved by wet chemical etching in 5% wt. orthophosphoric acid at room temperature for 3 h (sample Ox), also resulting in a noticeable increase in pore size from the starting average pore diameter of 35 nm to around 48 nm.

ALD SiO_2_ coating was carried out in a thermal ALD reactor (Savannah 100, Cambridge Nanotech, Waltham, MA, USA), operated in exposure mode. The precursors employed for SiO_2_ deposition were APTES (100 °C), water (60 °C) and ozone (20 °C) for SiO_2_, whereas the reaction chamber was kept at 180 °C throughout the ALD processes. A constant flow of 50 sccm of high purity argon gas was employed as both, purge and carrier gas. Additional details of the ALD deposition sequence can be found elsewhere [7,12]. A total of 80 deposition cycles were performed, being the ALD process stopped after an additional APTES pulse (sample Ox+SiO_2_/APTES) or after an APTES/H_2_O/O_3_ pulse sequence (sample Ox+SiO_2_/APTES/O_3_).

### 2.2. SEM Characterization

The morphology of the nanoporous alumina membranes, including top, bottom and cross-sections views, was studied by scanning electron microscopy (SEM) in a JEOL-5600 Scanning Microscope (Akishima, Tokyo, Japan). Prior to SEM observation, the membranes were coated with a thin gold layer deposited by sputtering (Polaron SC7620, Quorum Technologies, Laughton, UK), in order to improve their electrical conductivity. The geometrical parameters (nanopore size and interpore distance) were measured by computer assisted image analysis using ImageJ (v 1.51j8) [30].

### 2.3. Chemical Surface Characterization

X-ray photoelectron spectroscopy (XPS), a non-destructive surface sensitive technique, was used to determine the quantity and chemical state of elements present in the surface (or near the surface, around 2–8 nm depth) of the studied samples. XPS spectra were obtained with a Physical Electronics Spectrometer VersaProbe II (Physical Electronics, Chigasaki, Japan) using monochromatic Al-K_α_ radiation (49.1 W, 15 kV and 1486.6 eV) considering a circular area of 200 μm in diameter, using a hemispherical multichannel detector. The residual pressure in the analysis chamber was maintained below 5 × 10^−7^ Pa during data acquisition. Spectra were recorded with a constant pass energy value at 29.35 eV, at a take-off angle of 45 °C, and each spectral region was scanned several times to reduce the noise ratio. Binding energies (B.E. accurate ± 0.1 eV) were determined with respect to the position of the adventitious C 1s peak at 285.0 eV. PHI ACCESS ESCA-V8.0 F software package was used for data acquisition and analysis [31].

### 2.4. Electrochemical Impedance Spectroscopy Measurements

Electrochemical Impedance Spectroscopy (EIS) is an alternating current (a.c.) technique commonly used for electrical characterization of both homogeneous (solid and liquids) or heterogeneous systems commonly used for electrical characterization of both homogeneous (solid and liquid) or heterogeneous systems [32,33]. EIS measurements were carried out in an electrochemical test-cell as that described in previous papers [33,34], which basically consists of two glass compartments filled with an electrolyte solution of the same concentration, separated by two silicone-rings where the sample (or membrane) was sandwiched, and two electrodes, one into each compartment, connected to a Frequency Response Analyzer (FRA, Solartron 1260, Farnborough, UK). Measurements were performed with NaCl solutions in the system: electrode/NaCl solution (C)//sample//NaCl/solution (C)/electrode, for three different concentration values: C = 2 × 10^−3^ M, 6 × 10^−3^ M and 1 × 10^−2^ M. 100 different data points for frequency ranging between 1 Hz and 10^7^ Hz, at maximum voltage of 0.01 V, were recorded. ZView 2 data analysis program (Scribner, Southern Pines, NC, USA) was used for electrical parameters determination.

EIS measurements give quantitative/qualitative information related to charge movement/adsorption from experimental data using equivalent circuits as models [33,34,35] and, under certain conditions, the analysis of EIS diagrams allows us the estimation of different membrane system contributions (membrane, electrolyte and membrane/solution interface). The impedance (Z = Z_real_ + j Z_img_) is a complex number, with real (Z_real_) and imaginary (Z_img_) parts, which can be expressed for homogeneous systems (a unique relaxation process) as functions of electrical resistance (R) and capacitance (C) of the analysed sample:

Z_real_ = R/[1 + (ωRC)^2^] (1)

Z_img_ =−ωR^2^ C/[1 + (ωRC)^2^] (2)

where ω = 2πf is the angular frequency. The analysis of the impedance data is usually performed in the complex plane by analysing the Nyquist plot (−Z_img_ versus Z_real_), where a parallel association of a resistance and a capacitance (RC circuit) corresponds to a semi-circle which intercepts the Z_real_ axis at R_∞_ (ω→∞) and R_o_ (ω→0), being R = 0.5(R_o_ −R_∞_) the electrical resistance of the system, while the maximum of the semi-circle occurs at a frequency such that ωRC = 1 [32]. In the case of non-homogeneous systems Nyquist plot exhibits a depressed semicircle (two or more different relaxation times), and an equivalent capacitance is then considered [32].

### 2.5. Optical Characterizations

Optical characterization was performed by analysing transmittance, photoluminescence (PL) and spectroscopic ellipsometry (SE) curves.

Light transmission spectra were recorded for wavelengths ranging between 250 nm and 2000 nm, with a Varian Cary 5000 spectrophotometer (Agilent Technologies, Santa Clara, CA, USA) provided with an integrating sphere of Spectralon. Measurements were performed covering samples surface with a mask which exhibits an open area of 0.5 cm × 0.5 cm.

Samples photoluminescence (PL) was measured with a Photoluminiscence Microscope from HORIBA Scientific (LabRam PL Microscope, Horiba Jobin Yvon, Kyoto, Japan), using a 325 nm laser as excitation light source with a beam power of 0.28 mW in the range from 325 to 900 nm.

Spectroscopic ellipsometry (SE) measurements were carried out with a spectroscopic ellipsometer (Sopra-Semilab GES-5E, Budapest, Hungary) at an incident angle of 70° and wavelength ranging between 250 nm and 1700 nm, covering a wide range of visible and near infrared (NIR) regions. Measurements were directly performed on the samples without employing any other substrate.

## 3. Results and Discussion

### 3.1. SEM Characterization

**Figure 2:**
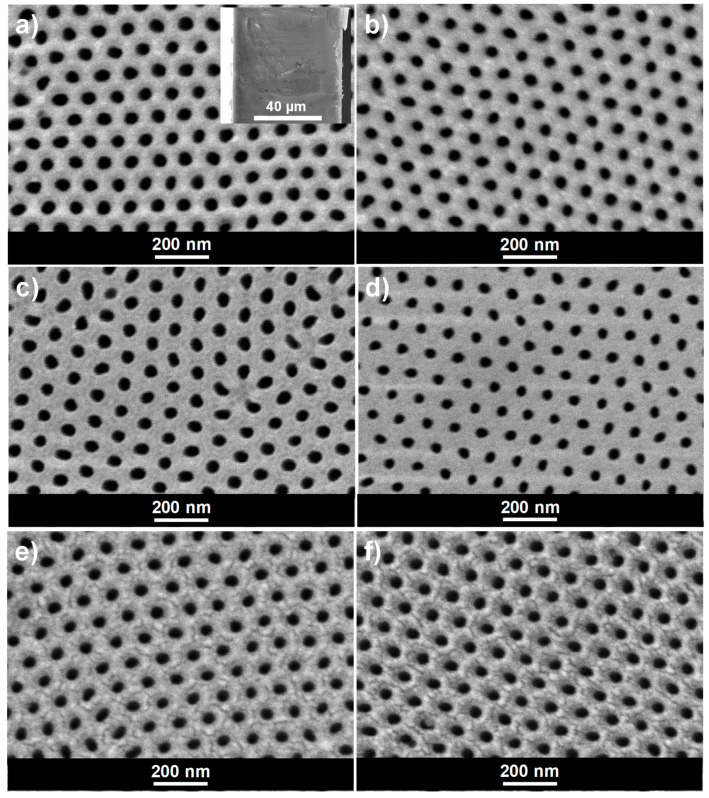
SEM micrographs of nanoporous alumina membrane (**a**) as produced (Ox sample), top view and cross section (inset); (**b**) after ALD SiO_2_ deposition (Ox+SiO_2_), top view; (**c**) as produced (Ox sample), bottom view (**d**) after ALD SiO_2_ deposition (Ox+SiO_2_), bottom view (**e**) after SiO_2_/APTES ALD cycle (Ox+SiO_2_/APTES), and (**f**) after SiO_2_/APTES/O_3_ ALD cycle (Ox+SiO_2_/APTES/O_3_).

NPAS samples synthesized by the two-step anodization method display a highly ordered pore arrangement with hexagonal symmetry at both, top and bottom sides, as it is evidenced by SEM images shown in Figure 2. Figure 2a,c correspond respectively to the upper and lower surfaces of the as-produced nanoporous alumina sample (Ox), being the corresponding cross-section presented in the inset, whereas images in Figure 2b–f were taken after: Figure 2b,c the ALD deposition of 80 cycles of SiO_2_ (Ox+SiO_2_ sample), Figure 2e plus an additional APTES exposure (Ox+SiO_2_/APTES) and, finally, Figure 2f plus an additional O_3_ pulse (Ox+SiO_2_/APTES/O_3_).

**Figure 3:**
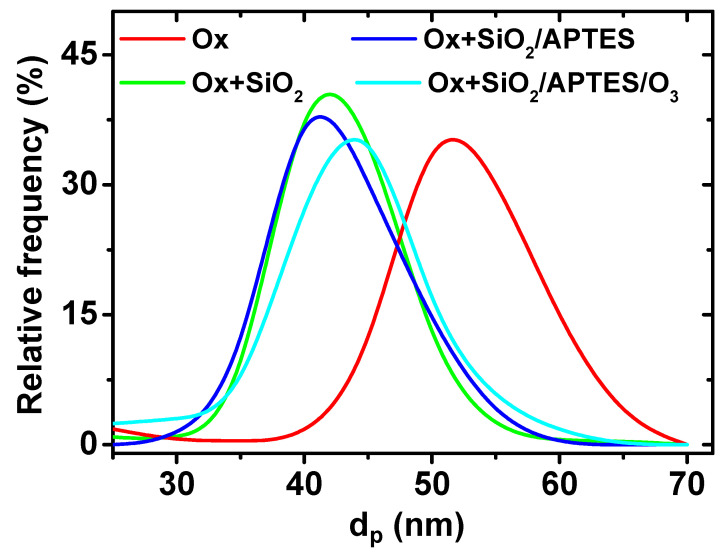
Pore diameter distribution obtained by image analysis of nanoporous alumina membranes before (Ox) and after SiO_2_ (Ox+SiO_2_), SiO_2_/APTES (OX+ SiO_2_/APTES) and SiO_2_ (OX+ SiO_2_)/APTES/O_3_ ALD deposition.

The reduction in the average pore diameter as a result of the SiO_2_ coating layer becomes evident from the analysis of the pore diameter distribution displayed in Figure 3, by comparison of samples Ox and Ox+SiO_2_. The average pore diameter was reduced from 48 ± 6 nm down to 40 ± 6 nm as a result of the ALD SiO_2_ coating, which indicates a thickness of 4 nm for the SiO_2_ layer. At the same time, SEM images evidence that differences in the average pore diameter among Ox+SiO_2_, Ox+SiO_2_/APTES and Ox+SiO_2_/APTES/O_3_ samples are practically negligible and, consequently similar pore size/porosity is assumed for these three samples. The interpore distance, which remains unaffected by the ALD coating, takes a value of 105 nm for all the indicated samples. Membranes thickness, evaluated from SEM cross section images (see inset in Figure 2a, takes a value of approximately 60 microns for all the samples.

### 3.2. Chemical Surface Characterization

**Figure 4:**
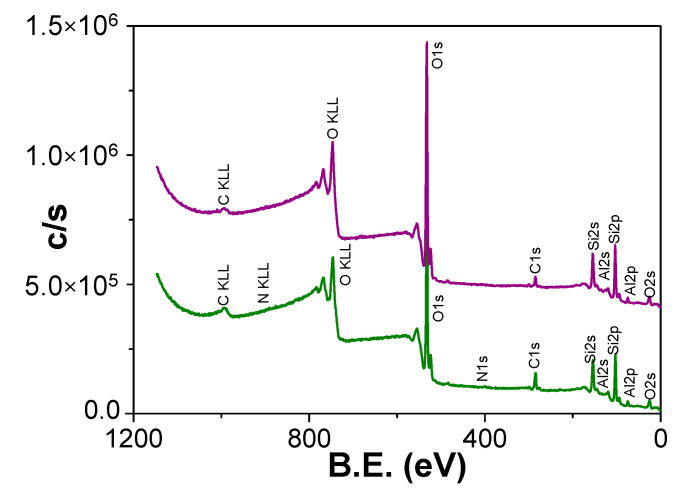
Survey XPS spectra of Ox+SiO_2_/APTES (green line) and Ox+SiO_2_/APTES/O_3_ (purple line) samples.

Chemical surface characterization of Ox+SiO_2_/APTES and Ox+SiO_2_/APTES/O_3_ NPASs was performed by analysing the XPS spectra. Figure 4 shows the survey spectra obtained for each sample, where the characteristic photoemission lines corresponding to C, O, N, Si and Al atoms are labelled

**Figure 5:**
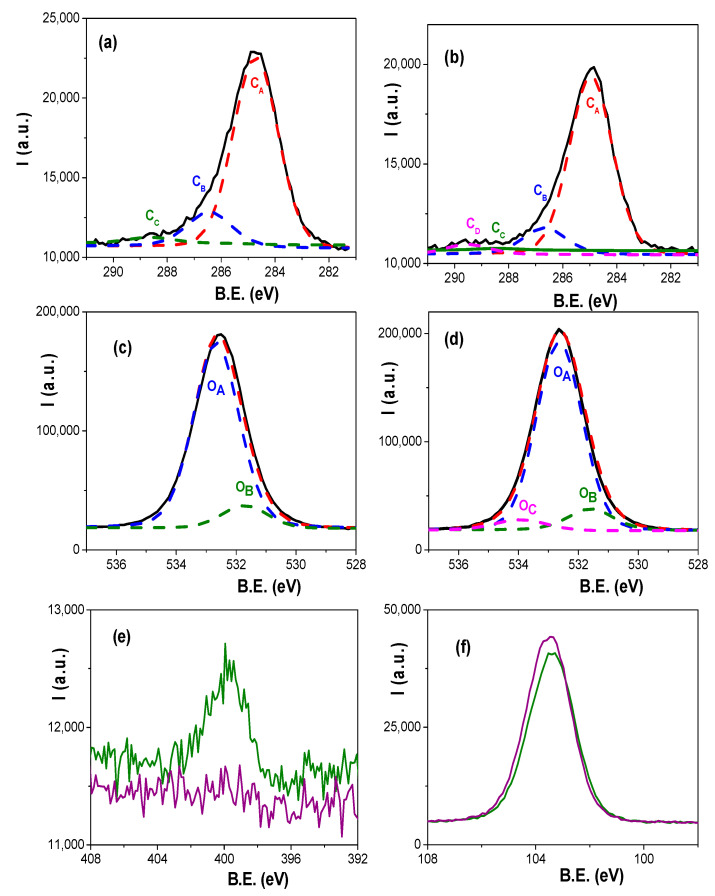
Carbon core level spectra (black solid line) and its deconvolution for: (**a**) Ox+SiO_2_/APTES: C_A_, dashed red line; C_B_, dashed blue line; C_c_, dashed green line; (**b**) Ox+SiO_2_/APTES/O_3_: C_A_, dashed red line; C_B_, dashed blue line; C_c_, dashed green line; C_D_, dashed magenta line. Oxygen core level spectra (black solid line) and its deconvolution for: (**c**) Ox+SiO_2_/APTES sample: O_A_, dashed blue line; O_B_, dashed green line; (**d**) Ox+SiO_2_/APTES/O_3_ sample: O_A_, dashed blue line; O_B_, dashed green line; O_c_, dashed magenta line. Experimental values: red solid line; fitted values: dashed red line. (**e**) Nitrogen and (**f**) Silicon core level spectra for Ox+SiO_2_/APTES (green solid line) and Ox+SiO_2_/APTES/O_3_ (purple solid line) samples.

Atomic concentration percentage (A.C. %) of each element was obtained by the corresponding areas, and their values are indicated in Table 1, where the percentages of Si and Al (element associated to the nano-support structure) are also showed; in fact, the relatively low value of this latter element is an indication of adequate coating process. For both samples, carbon core level signal (black solid line in Figure 5a for Ox+SiO_2_/APTES sample and in Figure 5b for Ox+SiO_2_/APTES/O_3_ one) shows a clear peak at 285.0 eV B.E. associated to aliphatic carbon, but other small shoulders at higher binding energy values corresponding to other carbon links are also detected [36], which will be discussed in the next paragraph as well as the slight differences in oxygen core level signal obtained for both samples (black solid line in Figure 5c,d for Ox+SiO_2_/APTES and Ox+SiO_2_/APTES/O_3_, respectively) are also indicative of other contributions different from the high intensity peak at B.E. of 532.6 eV associated to Si–O/SiO_2_ links [36]. On the other hand, a clear peak at a B.E. of 399.5 eV can be observed in the nitrogen core level spectra (Figure 5e) for the Ox+SiO_2_/APTES sample but, as expected, the Ox+SiO_2_/APTES/O_3_ one does not show any nitrogen contribution, which demonstrates the total elimination of amino groups by O_3_ treatment; in the case of silicon core level spectra (Figure 5f) both samples show similar curves, with a peak at the B.E. of 103.4 eV.

As it was shown above, the asymmetry of carbon core level spectra is an indication of different carbon links, and three different contributions (represented by dashed lines) are pointed out in Figure 5a for Ox+SiO_2_/APTES sample but four in Figure 5b for Ox+SiO_2_/APTES/O_3_ one: the peak C_A_ (at 285.0 eV B.E.) of higher intensity is associated to C–C or C–H links, while the small shoulders C_B_ (at 286.6 eV B.E.) and C_C_ (at 288.9 eV B.E.) are associated to CO/CN/CSi and C–O bonds, respectively [36]; moreover, another shoulder C_D_ (at 289.4 eV B.E.) associated to the O–C=O bond was also determined in the case of the Ox+SiO_2_/APTES/O_3_ sample. The corresponding area percentage and atomic concentration percentage determined for each sample as well as the binding energy values and links are collected in Table 2A.

The O 1s core level spectra for the studied samples shown in Figure 5c,d exhibits a very intense peak at a B.E. of 532.6 eV (O_A_), associated to Si–O/SiO_2_ links, but also a small shoulder at 531.6 eV (O_B_) which corresponds to Al–O bond [36]; however, the Ox+SiO_2_/APTES/O_3_ sample also shows another shoulder at a B.E. of 533.9 eV associated to C–O link [36]. Comparison of experimental (black solid line) and fitted (dashed red line) oxygen values are also drawn in Figure 5c,d while area percentage and atomic concentration percentage of the different contributions determined for each sample are also indicated in Table 2B, as well as the corresponding binding energy values and links. In this context, it should be indicated the good concordance existing between the aluminum atomic concentration % directly obtained from Al core level area for Ox+SiO_2_/APTES and Ox+SiO_2_/APTES/O_3_ samples indicated in Table 1 (4.0 and 3.9%), and those determined from oxygen A.C. % for Al–O link shown in Table 2B, taking into account, Al_2_O_3_ stoichiometry.

### 3.3. Electrochemical Impedance Spectroscopy Measurements

**Figure 6:**
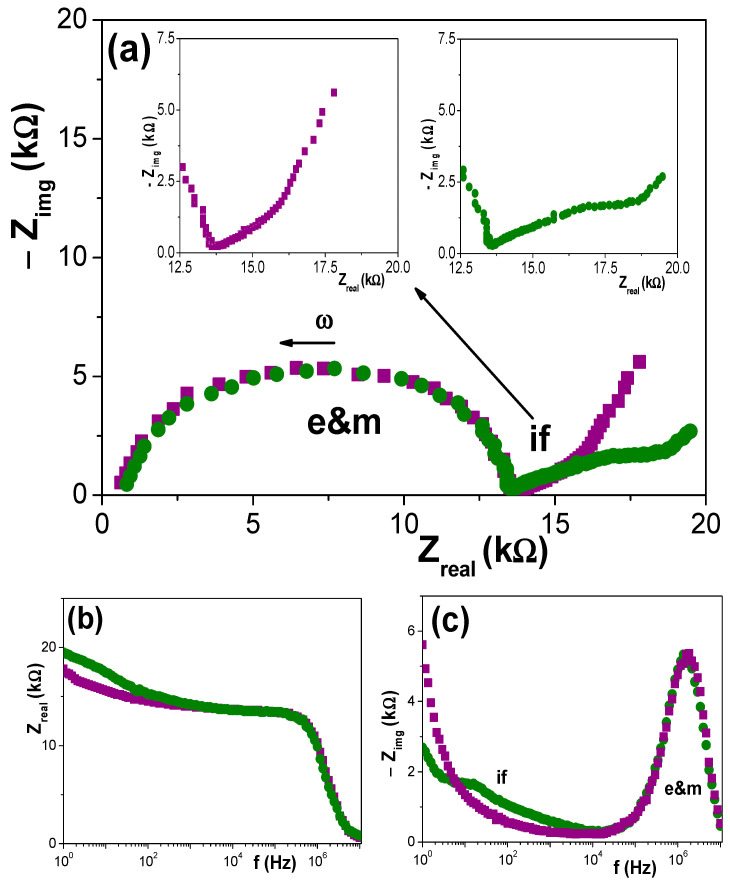
Comparison of impedance plots for Ox–SiO_2_/APTES (●) and Ox–SiO_2_/APTES/O_3_ (■) samples measured with 0.002 M NaCl solution. (**a**) Nyquist plot (−Z_img_ vs. Z_real_); (**b**) Bode plot (Z_real_ vs. frequency) and (**c**) Bode plot (−Z_img_ vs. frequency).

Once the chemical differences on the surface of Ox–SiO_2_/APTES and Ox–SiO_2_/APTES/O_3_ samples were established by XPS analysis, information on the effect of surface modification on diverse physicochemical parameters of interest for different sample applications (membrane, biochemical sensors, optical sensors,…) was determined. Information on electrochemical parameters involved in the transport of charged species (ions or macromolecules) across membranes is usually obtained by electrochemical measurements [7,24,37,38,39]. In particular, electrochemical impedance spectroscopy (EIS) is a technique commonly used in the electrical characterization of membranes in “working conditions”, that is, in contact and/or filled with electrolyte solutions, since it provides quantitative and qualitative information by the fitting of the impedance data using equivalent circuits as models or comparing the impedance diagrams determined for different systems [33,34,35]. Figure 6a shows the Nyquist plot (−Z_img_ vs. Z_real_) obtained for Ox–SiO_2_/APTES and Ox–SiO_2_/APTES/O_3_ membranes in contact with a 0.002 M NaCl solution, where two different relaxation processes can be observed: (i) one relaxation is associated to the membrane (with the nanopores filled with the electrolyte solution) plus the electrolyte solution placed between the membrane surface and the electrodes (denominated e&m), and (ii) the other relaxation corresponds to the electrolyte/membrane interface (if) [34,35,40,41]. As it can be observed, the e&m contribution is practically coincident for both membranes, which is an indication of their geometrical similarity and the very low electrical effect caused by APTES or APTES/O_3_ modifications in the bulk membrane, but clear changes exist in the electrolyte/membrane interfacial region in agreement with superficial changes proposed in the scheme indicated in Figure 1 and obtained by XPS analysis. These two aspects can also be observed in the Bode plots shown in Figure 6b (Z_real_ vs. frequency) and Figure 6c (−Z_img_ vs. frequency).

**Figure 7:**
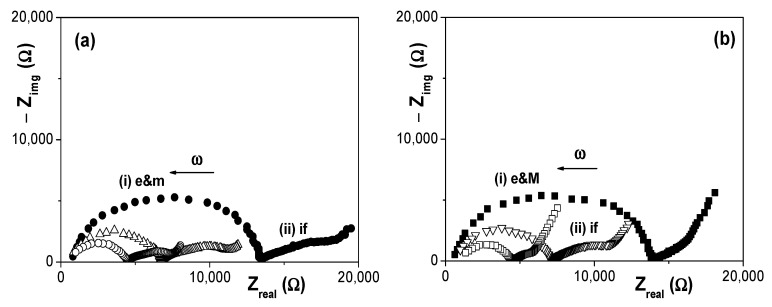
Comparison of Nyquist plots obtained for three different NaCl solution concentrations. (**a**) Ox–SiO_2_/APTES membrane system: (●) 0.002 M NaCl solution, (Δ) 0.006 M NaCl solution and (o) 0.01 M NaCl solution. (**b**) Ox–SiO_2_/APTES/O_3_ membrane system: (■) 0.002 M NaCl solution, (▽) 0.006 M NaCl solution and (□) 0.01 M NaCl solution.

The effect of electrolyte solution concentration on EIS data is clearly shown in Figure 7, where a comparison of Nyquist plots obtained for both membranes at the three NaCl concentrations studied (2 × 10^−3^ M, 6 × 10^−3^ M and 1 × 10^−2^ M) is presented. As it can be observed, the membrane plus electrolyte e&m contribution, which corresponds to a slightly depressed semicircle as a result of juxtaposition of two relaxation process (one associated to the membrane and other to the electrolyte) is significantly dependent on solution concentration and, logically it decreases with the increase of charges (ions) in solution, but solution concentration also seems to affect the interfacial part.

**Figure 8:**
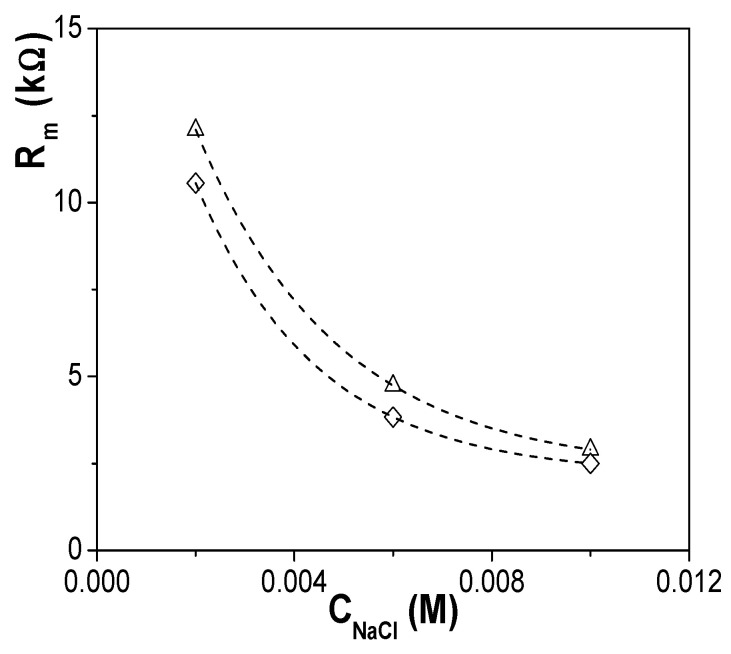
Membrane resistance dependence with NaCl concentration for both Ox–SiO_2_/APTES (◊) and Ox–SiO_2_/APTES/O_3_ (Δ) membranes.

EIS data for the two membranes plus electrolyte e&m contribution were fitted to a series combination of two contributions: (a) a parallel association of a resistance and a capacitor for the electrolyte solution (equivalent circuit: R_e_C_e_); (b) a parallel association of a resistance and a constant phase element or equivalent capacitor (CPE: Q(ω) = Y_o_(ω)^n^, for the membrane contribution (equivalent circuit: R_m_Q_m_)) [32,42]. Figure 8 shows the concentration dependence of R_m_ obtained for the Ox+SiO_2_/APTES and the Ox+SiO_2_/O_3_ membranes, where slight differences (around 15%) between both samples can be observed. Assuming similarity in pore-size/porosity of both alumina supports, in agreement with SEM results, R_m_ differences might be associated to the particular electrical surface characteristic related to the different modifying layer of each membrane.

### 3.4. Optical Characterization

Optical techniques are of great interest in the characterization of materials due to their non-invasive and non-destructive character. In particular, spectroscopic ellipsometry has already demonstrated their suitability for characterization of nanoporous alumina membranes with different pore size, porosity or structure [43], but also for biosensors characterization [44], while light transmission spectra allows estimation of differences in both geometrical parameters (pore-size/porosity) and surface material of analyzed samples [8,12]. Moreover, a particular property of nanoporous alumina structures (NPASs) obtained by the two-step anodization method such as their potoluminescence (PL) character, which is associated to the ionized oxygen vacancies and the carboxylate from the electrolyte solution incorporated into the NPAS during the fabrication process has also been reported [45,46,47].

**Figure 9:**
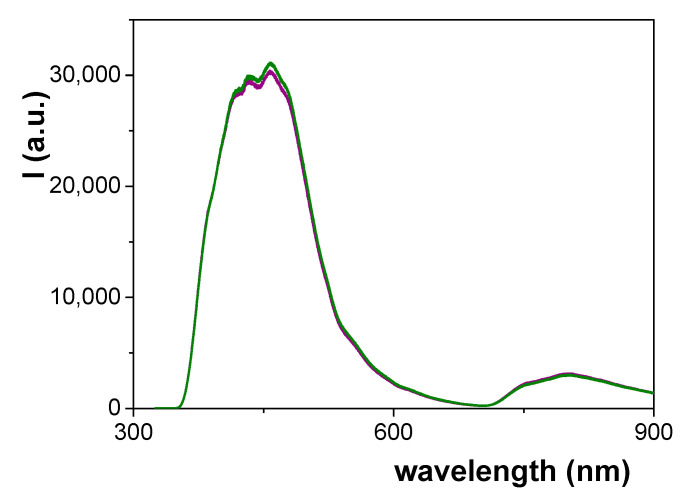
Photoluminescence spectra for Ox–SiO_2_/APTES sample (green line) and Ox-SiO_2_/APTES/O_3_ sample (purple line).

Figure 9 shows a comparison of the PL spectra obtained for the Ox+SiO_2_/APTES and Ox+SiO_2_/APTES/O_3_ membranes, where the similarity of the curves obtained for both samples, which is associated to alumina support bulk phase as it was indicated above, is very significant. In fact, the PL spectra of nanoporous alumina structures seems to be related to the incorporation of impurities to the structure of anodic alumina, which is strongly dependent on the fabrication parameters (anodization voltage, electrolyte, thermal treatment,…) [48,49]. In particular, an increase in the intensity of the PL curve as well as a shift of the wavelength value associated to the maximum of the curve with the increase of pore size have already been reported for membranes fabricated using oxalic acid as electrolyte [50]. Consequently, the likeness of both PL curves can be considered as a confirmation of alumina support similarity.

**Figure 10:**
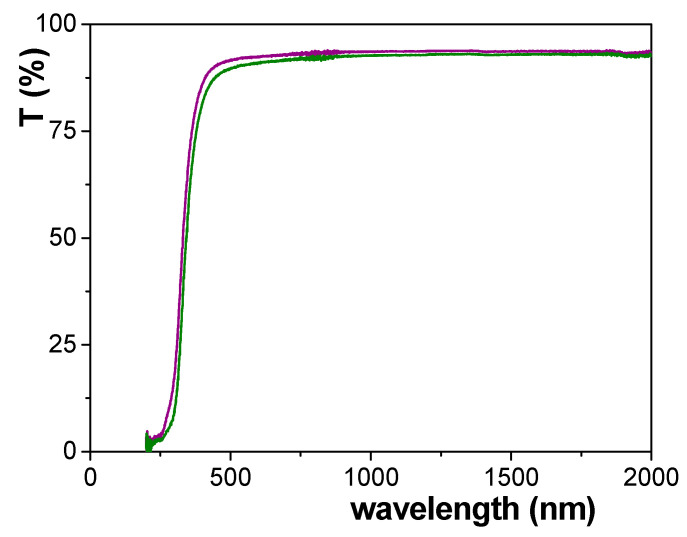
Transmission spectra for Ox–SiO_2_/APTES sample (green line) and Ox–SiO_2_/APTES/O_3_ sample (purple line).

A comparison of transmission spectra for both types of NPASs, Ox–SiO_2_/APTES and Ox–SiO_2_/APTES/O_3_ samples, is shown in Figure 10, where two main aspects, the slight difference between both curves for the whole range of wavelength and the high transparency of both analyzed samples, can be observed. With respect to this latter point, a comparison between the transmittance spectra for diverse alumina-based structures obtained after surface coverage by ALD technique with different metal oxides, and consequently with different material surface but similar geometrical parameters, has been already reported in previous works [8,12] and it is shown as Supplementary Information (Figure S1). These results demonstrate the significant effect of sample material on light transmittance, showing values of around 90% even for samples/membranes with reduced free volume (10–13 nm pore size and 4–6% porosity). This higher influence observed in the case of those nanoporous membranes is due to the peculiar optical properties exhibited by anodic alumina together with these coming from the different ALD layers further deposited on it, which become them in outstanding materials for optical and photonic applications [51].

In the case of uncoated NPASs, noticeable changes in transmittance spectra have been reported among samples synthesized under modulated anodic electrochemical conditions, i.e., temperature, anodic voltage, etc., but these modifications are related to the formation of Distributed Bragg reflectors (DBR) due to periodic modulation of the synthesis conditions, which in turn results in periodic variations in the morphological parameter of alumina, namely pore size and porosity [52,53].

With respect to the differences exhibited by the transmittance curves of the analyzed samples, the following transparency percentage values at three given wavelengths, 400 nm, 800 nm and 2000 nm, were obtained: 81.7%, 92.4% and 92.7% for Ox–SiO_2_/APTES membrane, but 86.1%, 93.4 and 93.6% for Ox–SiO_2_/APTES/O_3_ one. These results indicate a difference between both samples for both extremes of the visible region of around 5% and 1%, respectively, but a practically constant difference of 1% for the near infrared region (800–2000 nm), which seems to indicate that transmission difference is more probably related to membrane material change due to APTES or APTES/O_3_ modification than to geometrical effects. On the other hand, band gap value for Ox–SiO_2_/APTES and Ox–SiO_2_/APTES/O_3_ samples hardy differ one from the other (298 nm and 286, respectively), and they are similar to that determined for a SiO_2_-covered nanoporous alumina structure obtained also using 0.3 M oxalic acid solution and the same anodization voltage in the two-step process (288 nm [8,12]); although these values are higher than that referred for pure SiO_2_ (120–155 nm, [54]) due to the presence of other modifying elements and/or impurities on samples surfaces, according to XPS results.

Spectroscopic ellipsometry (SE) is an optical technique used for the characterization of thin films and membranes able to determine changes associated to bulk phase or surface modifications [8,12,55,56]. SE measures changes in light polarization due to its reflection/transmission across a solid structure by considering two characteristic parameters, angles Ψ and Δ, which are related with differential changes in amplitude and phase between light waves through the Fresnel reflection coefficients ratio, r_s_ and r_p_, of polarized light (s for perpendicular and p for parallel) by [57]:

tan(Ψ)e^i^^Δ^ = r_p_/r_s_ (3)

**Figure 11:**
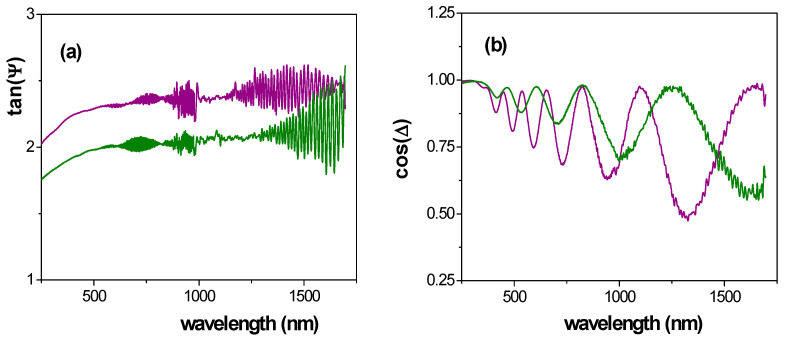
Wavelength dependence of: (**a**) tanΨ, (**b**) cosΔ, for Ox–SiO_2_/APTES (green line) and Ox-SiO_2_/APTES/O_3_ (purple line) samples.

A comparison of wavelength dependence of both experimental parameters for Ox–SiO_2_/APTES and Ox–SiO_2_/APTES/O_3_ samples is shown in Figure 11, where the interference fringes in tan ψ exhibited by both samples at high wavelength values are an indication of high sample transparency [58], in agreement with transmittance results already indicated. On the other hand, the oscillatory character of cos(Δ) has already been reported in the literature for anodized nanoporous alumina structures or membranes and it is related with sample pore size, surface impurities and roughness [58,59,60], while the effect of material surface on tan(Ψ) values for ALD modified NPASs has already been reported [8,12].

**Figure 12:**
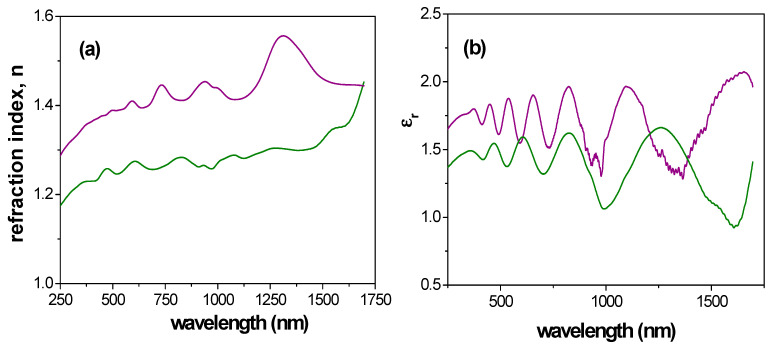
Wavelength dependence of: (**a**) refractive index; (**b**) real part of dielectric constant. Sample Ox+SiO_2_/APTES: solid green line, Ox+SiO_2_/APTES/O_3_: solid purple line.

From experimental Ψ and Δ values, using a Cauchy dispersion model relation [61] to describe the dependence of the refractive index on the wavelength, different optical characteristic parameters such as the refractive index (n) or the dielectric constant (ε) (ε = (n + i·k)^2^, where k represents the extinction coefficient) can be determined [57]). Figure 12 shows a comparison of wavelength dependence for the refractive index (Figure 12a) and the real part of the dielectric constant (Figure 12b) for Ox–SiO_2_/APTES and Ox–SiO_2_/APTES/O_3_ samples, where differences depending on surface modification and optical regions are obtained. In fact, curves shape in visible region are rather similar, with higher variation in the near infrared region.

Average values of refraction index and dielectric constant for the whole range of wavelength as well as for visible and near infrared regions were determined for comparison reason, and these results are indicated in Table 3. A comparison of the <n> value shown in Table 3 with that referred for APTES indicates a reduction of 10% (n^APTES^ = 1.423 [62]), or 12% in the case of SiO_2_ thin film (n^SiO^^2^ = 1.46) [63]. On the other hand, ratio of refraction index and dielectric constant average values determined for both samples are practically independent of wavelength interval, being <n>^Ox+SiO2/APTES^/<n>^Ox+SiO2/APTES/O^^3^ ~ 0.9 and <ε_r_>^Ox+SiO2/APTES^/<ε_r_>^Ox+SiO2/APTES/O^^3^ ~ 0.8, proving evidence of surface effect difference associated to samples modification. Furthermore, the possibility of performing *in*-*situ* SE measurements during ALD deposition in both, custom-made and commercial devices, provides not only an accurate monitoring of the deposited film thickness and growth mechanisms, but also a powerful tool to determine film optical properties, including the possibility to tune at will some physical parameters of the samples such as the refraction index [64,65,66].

## 4. Conclusions

Deposition of SiO_2_ on a nanoporous alumina support by thermal ALD using a self-catalytic reaction based on 3-aminopropyltriethoxysilane (APTES), water, and ozone as precursors, in a three-step reaction sequence at low growth temperature, allows us to provide to the nanoporous support different surface functional groups (Ox+SiO_2_/APTES and Ox+SiO_2_/APTES/O_3_ samples), which might be of interest in different applications such as optical or electrochemical sensors, membranes or nanofluidic. XPS analysis was used for estimation of chemical surface modification, while spectroscopic ellipsometry measurements indicate changes in optical properties such as refractive index and dielectric constant, because of the difference in the surface functional groups, which enables in-situ monitoring ALD growth. Surface chemical modification also seems to affect interfacial effects when the studied samples are in contact with electrolyte solutions according to electrochemical spectroscopy results. However, surface modification hardly affects to characteristics of anodic alumina related to bulk support properties such as their photoluminescence and morphological parameters (pore diameter and porosity) taking into account the similarity exhibited by SEM micrographs and PL curves obtained for Ox+SiO_2_/APTES and Ox+SiO_2_/APTES/O_3_ samples.

## Figures and Tables

**Table 1:** Atomic concentration percentages of the elements found on samples surfaces *.

Sample	C (%)	O (%)	N (%)	Si (%)	Al (%)
Ox+SiO_2_/APTES	12.8	55.3	0.7	26.9	4.0
Ox+SiO_2_/APTES/O_3_	8.3	59.3	-	28.1	3.9

* Elements associated to impurities with A.C. % < 0.3 are not indicated.

**Table 2:** Binding energy (B.E.) of the different links, area percentages (*cursive*) and A.C. % after carbon (A) and oxygen (B) signals deconvolution.

(A)		-	-	-	-
Sample		C_A_ (%)	C_B_ (%)	C_C_ (%)	C_D_ (%)
B.E. (eV)		285.0	286.6	288.9	289.4
-		CC/CH	CO/CN/CSi	C=O	O–C=O
Ox+SiO_2_/APTES	*Area %*	*81.3*	*16.43*	*2.2*	*-*
	A.C. %	10.4	2.1	0.3	-
Ox+SiO_2_/APTES/O_3_	*Area %*	*81.7*	*13.1*	*0.7*	*4.5*
	A.C. %	6.8	1.1	0.06	0.37
**(B)**		**-**	**-**	**-**	**-**
**Sample**		**O_A_ (%)**	**O_B_ (%)**	**O_C_ (%)**	
B.E. (eV)		531.6	532.6	533.9	
-		Al–O	Si–O/SiO_2_/O–C=O*	O*–C=O	
Ox+SiO_2_/APTES	*Area %*	*10.3*	*89.7*	*-*	
	A.C. %	5.7	49.6	-	
Ox+SiO_2_/APTES/O_3_	*Area %*	*10.5*	*84.5*	*5.0*	
	A.C. %	6.2	50.1	3.0	

**Table 3:** Average values of the refraction index, <n>, and the real part of dielectric constant, <ε_r_>, for different wavelength intervals.

Sample	Ox+SiO_2_/APTES	Ox+SiO_2_/APTES/O_3_
<n> 250–1700 nm	1.28 ± 0.04	1.43 ± 0.06
<ε_r_> 250–1700 nm	1.38 ± 0.20	1.71 ± 0.20
<n> 250–800 nm	1.25 ± 0.03	1.38 ± 0.04
<ε_r_> 250–800 nm	1.46 ± 0.07	1.72 ± 0.10
<n> 800–1700 nm	1.31 ± 0.04	1.46 ± 0.04
<ε_r_> 800–1700 nm	1.33 ± 0.23	1.71 ± 0.24

## Data Availability

Not Applicable.

## Supplementary Materials

The following are available online at https://www.mdpi.com/article/10.3390/ma14175052/s1, Figure S1: Transmission spectra for different nanoporous alumina-based structures with similar pore size (9 ± 2) nm and porosity (7 ± 2)%, but different coating layers.
