# Supplementary material for: Surface Modification of Nanoporous Anodic Alumina during Self-Catalytic Atomic Layer Deposition of Silicon Dioxide from (3-Aminopropyl)Triethoxysilane
Source: Materials (Basel). 2021 Sep 3;14(17):5052. doi: 10.3390/ma14175052 (PMC8434165; doi:10.3390/ma14175052)
Supplement: Supplementary file 1 [file materials-14-05052-s001.zip › materials-1332005-supplementary.pdf]

materials

MDPI

Supplementary Materials

Surface Modification of Nanoporous Anodic Alumina during Self-Catalytic Atomic Layer Deposition of Silicon Dioxide from (3-Aminopropyl)Triethoxysilane

Ana Silvia González [1,*], Víctor Vega [2], Ana Laura Cuevas [3], María del Valle Martínez de Yuso [4], Víctor M. Prida [1] and Juana Benavente [5]

1 Departamento de Física, Facultad de Ciencias, Universidad de Oviedo, E-33007 Oviedo, Spain; vmpp@uniovi.es
2 Laboratorio de Membranas Nanoporosas, Servicios Científico-Técnicos, Universidad de Oviedo, E-33006 Oviedo, Spain; vegavictor@uniovi.es
3 Unidad de Nanotecnología, SCBI Centro, Universidad de Málaga, E-29071 Málaga, Spain; analaura.cuevas@uma.es
4 Servicios Centrales de Investigación, Universidad de Málaga, E-29071 Málaga, Spain; mvyuso@uma.es
5 Departmento de Física Aplicada I, Facultad de Ciencias, Universidad de Málaga, E-29071 Málaga, Spain; j_benavente@uma.es
* Correspondence: gonzalezgana@uniovi.es

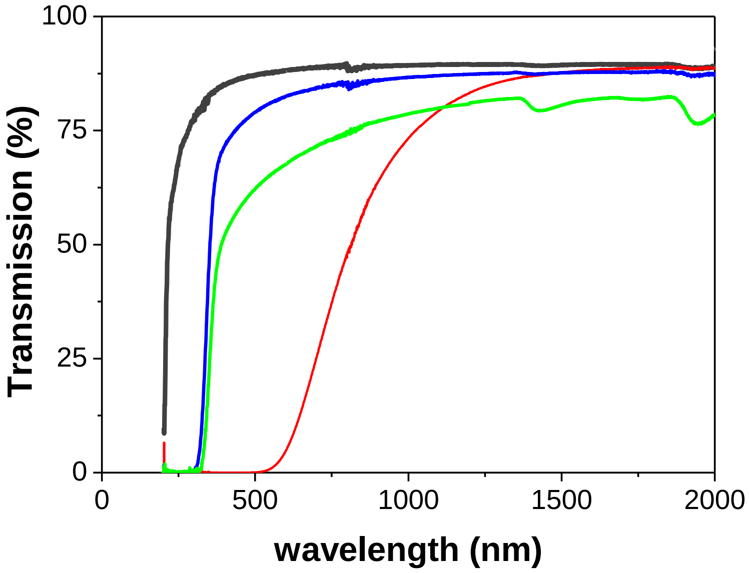

Figure S1. Transmission spectra for different nanoporous alumina-based structures with similar pore size (9 ± 2) nm and porosity (7 ± 2)%, but different coating layers: Al_2O_3 layer (black line), Fe_2O_3 layer (red line), TiO_2 layer (blue line) and TiO_2 layer after bovine serum albumin coverage (green line) [1,2].

References

1. Cuevas, A.L.; Martínez de Yuso, M.V.; Gelde, L.; Gonzalez, A.S.; Vega, V.; Prida, V.M., Benavente, J. Chemical, optical and transport characterization of ALD modified nanoporous alumina based structures. *J. Ind. Eng. Chem.* **2020**, *91*, 139–148.
2. Gelde, L.; Cuevas, A.L.; Benavente, J. Influence of pore-size/porosity on ion transport and static BSA-fouling for TiO_2-covered nanoporous alumina membranes. *Appl. Sci.* **2021**, *11*, 5687.
